# Antitumor Effects of Vitamin D Analogs on Hamster and Mouse Melanoma Cell Lines in Relation to Melanin Pigmentation

**DOI:** 10.3390/ijms16046645

**Published:** 2015-03-24

**Authors:** Tomasz Wasiewicz, Paulina Szyszka, Miroslawa Cichorek, Zorica Janjetovic, Robert C. Tuckey, Andrzej T. Slominski, Michal A. Zmijewski

**Affiliations:** 1 Department of Histology, Medical University of Gdańsk, Dębinki 1a, 80-211 Gdańsk, Poland; E-Mails: tomwasxp@poczta.onet.pl (T.W.); pszyszka@gumed.edu.pl (P.S.); 2 Department of Endocrinology and Internal Medicine, Medical University of Gdańsk, Dębinki 1a, 80-211 Gdańsk, Poland; 3 Department of Embryology, Medical University of Gdańsk, Dębinki 1a, 80-211 Gdańsk, Poland; E-Mail: cichorek@gumed.edu.pl; 4 Department of Dermatology, University of Alabama Birmingham, VA Medical Center, Birmingham, AL 35294, USA; E-Mails: zjanjeto@uthsc.edu (Z.J.); aslomins@uthsc.edu (A.T.S.); 5 School of Chemistry and Biochemistry, the University of Western Australia, Crawley, Perth, WA 6009, Australia; E-Mail: robert.tuckey@uwa.edu.au

**Keywords:** vitamin D, 1,25(OH)_2_D_3_, vitamin D analogs, secosteroids, lumisterol melanoma, melanin pigmentation, anti-melanoma effect

## Abstract

Deregulated melanogenesis is involved in melanomagenesis and melanoma progression and resistance to therapy. Vitamin D analogs have anti-melanoma activity. While the hypercalcaemic effect of the active form of Vitamin D (1,25(OH)_2_D_3_) limits its therapeutic use, novel Vitamin D analogs with a modified side chain demonstrate low calcaemic activity. We therefore examined the effect of secosteroidal analogs, both classic (1,25(OH)_2_D_3_ and 25(OH)D_3_), and novel relatively non-calcemic ones (20(OH)D_3_, calcipotriol, 21(OH)pD, pD and 20(OH)pL), on proliferation, colony formation in monolayer and soft-agar, and mRNA and protein expression by melanoma cells. Murine B16-F10 and hamster Bomirski Ab cell lines were shown to be effective models to study how melanogenesis affects anti-melanoma treatment. Novel Vitamin D analogs with a short side-chain and lumisterol-like 20(OH)pL efficiently inhibited rodent melanoma growth. Moderate pigmentation sensitized rodent melanoma cells towards Vitamin D analogs, and altered expression of key genes involved in Vitamin D signaling, which was opposite to the effect on heavily pigmented cells. Interestingly, melanogenesis inhibited ligand-induced Vitamin D receptor translocation and ligand-induced expression of *VDR* and *CYP24A1* genes. These findings indicate that melanogenesis can affect the anti-melanoma activity of Vitamin D analogs in a complex manner.

## 1. Introduction

Besides its classical role in calcium homeostasis and bone metabolism [1], the active form of Vitamin D_3_, 1α,25-dihydroxyvitamin D_3_ (1,25(OH)_2_D_3_), also known as calcitriol, demonstrates high therapeutic and prophylactic potential in osteoporosis, psoriasis and autoimmune diseases, to name just a few [2,3]. It also inhibits growth and induces the differentiation of multiple cell types [4,5]. Pleiotropic effects of 1,25(OH)_2_D_3_ include enhancement of DNA repair processes, protection against reactive oxygen species (ROS) and immunomodulation [6,7,8]. In humans, the majority of Vitamin D_3_ is synthesized by skin exposed to sunlight [9], where ultraviolet radiation B (280–320 nm) induces the photolysis of the B ring in 7-dehydrocholesterol (7-DHC). The resulting previtamin D_3_ isomerizes via a non-enzymatic reaction producing Vitamin D_3_ (cholecalciferol, (3β,5*Z*,7*E*)-9,10-secocholesta-5,7,10(19)-trien-3-ol). Further UV-irradiation of previtamin D3 produces lumisterol_3_ (9β,10α-cholesta-5,7-diene-3β-ol, L_3_), and tachysterol_3_ (*6E*-9,10-secocholesta-5(10),6,8-trien-3β-ol, T_3_). Vitamin D_3_ formation is influenced by both skin phenotype and the UVB dose [10,11,12]. UV radiation is also the main environmental factor involved in the development of skin cancer, including melanoma. The synthesis of melanin pigment in the skin represents a natural protective mechanism against UV-induced damage and carcinogenesis [13], but also limits Vitamin D_3_ synthesis [14].

Vitamin D_3_ is metabolized to the active form by the consecutive action of CYP2R1 or CYP27A1, catalyzing formation of 25(OH)D_3_ (25-hydroxyvitamin D_3_, calcifediol) in the liver, and CYP27B1, producing 1,25(OH)_2_D_3_ in the kidney [15,16,17,18]. 1,25(OH)_2_D_3_ is also synthesized locally by cells expressing the above enzymes, such as keratinocytes, dendritic cells, melanocytes, lymphocytes and different types of cancer cells [19,20,21,22,23,24]. The level of 1,25(OH)_2_D_3_ in the circulation is tightly regulated by 24-hydroxylase (CYP24A1), encoded by the *CYP24A1* gene [25]. Hydroxylation of 1,25(OH)_2_D_3_ at C24 results in a dramatic decrease in its biological activity and leads to further oxidation by CYP24A1 producing calcitroic acid which is excreted in the urine (for review see [26,27]).

To exert its biological activity, 1,25(OH)_2_D_3_ binds to the Vitamin D receptor (VDR). The VDR then heterodimerizes with the retinoid X receptor (RXR) and translocates to the nucleus where it regulates the expression of more than 900 genes, including ones involved in cell cycle progression, differentiation and apoptosis [28,29,30,31].

Despite its antiproliferative properties, the use of 1,25(OH)_2_D_3_ as a therapeutic agent at supra-physiological concentrations (above 50,000 units a day) is limited due to its hypercalcemic effects [4,12]. However, shortening or removal of the cholesterol-type side chain significantly reduces or abolishes the calcemic effects [32,33,34,35,36,37]. Furthermore, more than 3000 analogs of Vitamin D_3_ have been synthesized, with the biological activity of many of these still being extensively investigated, both as single agents (35) and in combination with other cytostatic compounds [38,39]. Recently, we discovered a new metabolic pathway of Vitamin D activation producing novel analogs, including 20-hydroxyvitamin D_3_ (20(OH)D_3_) (reviewed in [40,41]), which can operate *in vivo* [42], with the products showing strong antileukemic and anticancer activities *in vitro* (reviewed in [19,34,35,40]) while being noncalcemic and nontoxic *in vivo* [43,44]. We have already tested the *in vitro* activity of the major product of this pathway, 20(OH)D_3_, and its metabolites, finding that they show antiproliferative activity against cultured melanoma cells, acting as biased agonists on the VDR [43,45,46]. Importantly, 20(OH)D_3_ is noncalcemic and nontoxic *in vivo* at pharmacologically relevant doses [44], and is produced *in vivo* defining it as an endogenous/natural product [40,42].

Melanin pigmentation, a marker of melanocyte differentiation, affects cellular metabolism and behavior [13,47,48]. Although limited literature is available on the influence of melanin pigmentation on melanoma therapy, it has been demonstrated that melanogenesis shortens overall- and disease free-survival of patients with stage III and IV disease [49,50]. Furthermore, the presence and type of melanin pigment (eumelanin *vs*. pheomelanin) affects melanoma development in animal models [51]. In addition, inhibition of melanogenesis can sensitize human melanoma cells towards chemo-, immuno- and radio-therapy [52,53,54,55], as well as to treatment with Vitamin D analogs [35,36]. In highly pigmented melanomas, the efficacy of Vitamin D analogs decreases, probably as a result of diminished VDR expression [36,46,50].

In the current study, we investigated the cellular responses to classic and novel Vitamin D analogs in pigmented and non-pigmented hamster Ab, AbC1 and murine B16-F10 melanoma cell lines. These models were selected due to the ability to tightly regulate their melanin pigment production by the concentration l-tyrosine in the culture medium. Cells from primary cultures of transplantable tumors (Ab melanoma) and from established *in vitro* lines (AbC1 and B16-F10) were used for experiments.

## 2. Results

### 2.1. Moderate Pigmentation Enhances the Antiproliferative Effect of Vitamin D Analogs in Ab and B16-F10 Cells

Previous studies on human melanoma SKMEL-188 cells demonstrated that enhanced melanogenesis attenuated the antiproliferative activity of 20(OH)D_3_ which was associated with downregulation of VDR expression [46]. It had no effect on the antiproliferative effects of pD metabolites but enhanced the effects of lumisterol-like and tachysterol-like compounds [36]. To better understand the mechanism of differential action of Vitamin D and lumisterol metabolites in relation to the melanogenic pathway, we used two rodent (mouse and hamster) melanoma models in which melanin synthesis is inducible by melanin precursors [47,56,57].

**Figure 1:**
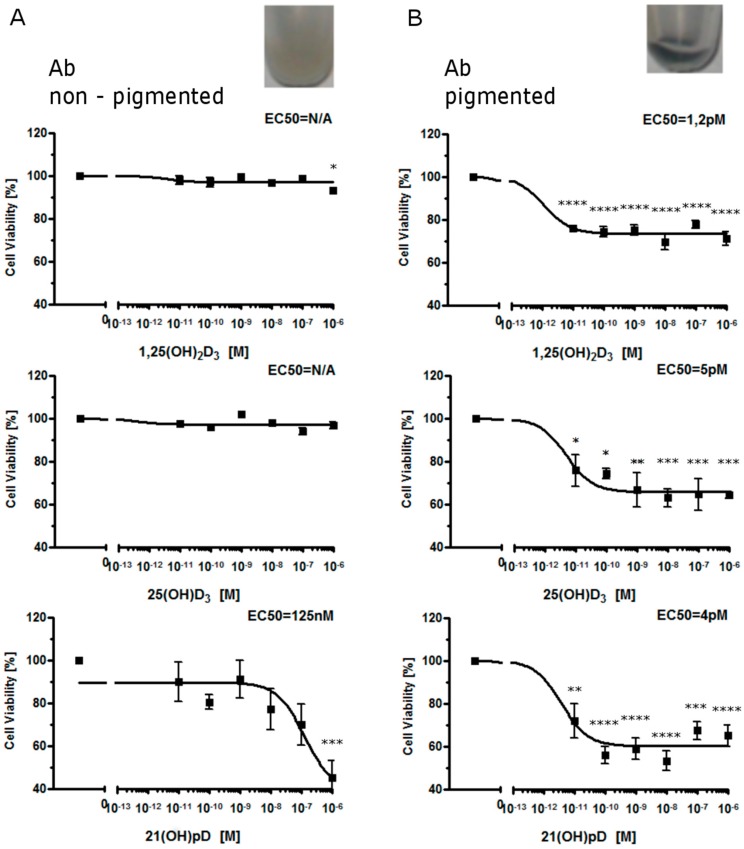
Inhibition of growth of hamster Ab melanoma cells by 1,25(OH)_2_D_3_, 25(OH)D_3_ and 21(OH)pD. Non-pigmented (**A**) and pigmented (**B**) Ab cells (grown as described in Materials and Methods section) were treated with serial dilutions of Vitamin D analogs (0.01–1000 nM). The experiment was repeated with similar results. The amelanotic and melanotic phenotypes are shown as inserts. Data are presented as *mean* ± SEM (*n* = 6). Statistical significance was estimated using *t*-test and presented as * *p* < 0.05, ** *p* < 0.005, *** *p* < 0.0005, **** *p* < 0.0001 *vs.* control.

**Figure 2:**
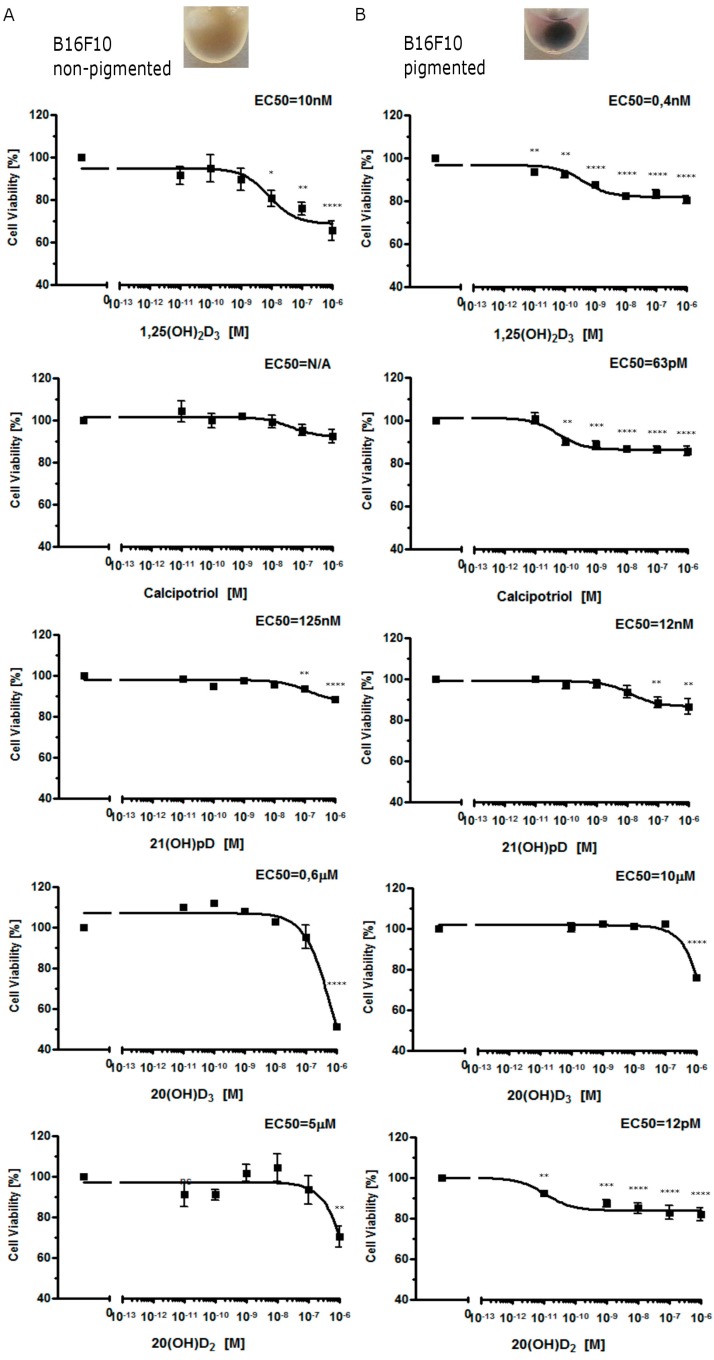
Growth inhibition of B16-F10 mouse melanoma cells by 1,25(OH)_2_D_3_, calcipotriol, 21(OH)pD, 20(OH)D_3_ and 20(OH)D_2_. Non–pigmented (**A**) and pigmented (**B**) B16-F10 cells were treated with serial dilutions of Vitamin D analogs (0.01–1000 nM). After 48 h the cells were subjected to a SRB test. The experiment was repeated with similar results. Examples of amelanotic and melanotic cells are shown as inserts above graphs. 1,25(OH)_2_D_3_ was used as a positive control. Data are presented as mean ± SEM for six independent measurements. Statistical significance was estimated using the *t*-test and presented as * *p* < 0.05, ** *p* < 0.005, *** *p* < 0.0005, **** *p* < 0.0001 *vs.* control.

In primary cultures of Ab cells freshly isolated from solid tumors, moderate melanin pigmentation sensitized cells towards 1,25(OH)_2_D_3_, 25(OH)D_3_ and the short side-chained 21(OH)pD analog. Interestingly, only 21(OH)pD demonstrated antiproliferative activity towards non-pigmented Ab primary cultures (Figure 1). In contrast, all compounds tested, including 1,25(OH)_2_D_3_, calcipotriol, 20(OH)D_3_, and 21(OH)pD, inhibited growth of non-pigmented murine B16-F10 cells (Figure 2). The sensitivity of pigmented Ab and B16-F10 cells towards Vitamin D analogs was higher in comparison to non-pigmented cells (Figure 1 and Figure 2) with one exception, the effect of 20(OH)D_3_ was similar for both pigmented and non-pigmented B16-F10 melanoma cells (Figure 2).

### 2.2. Vitamin D Analogs Inhibit the Growth of AbC1 Cells

**Figure 3:**
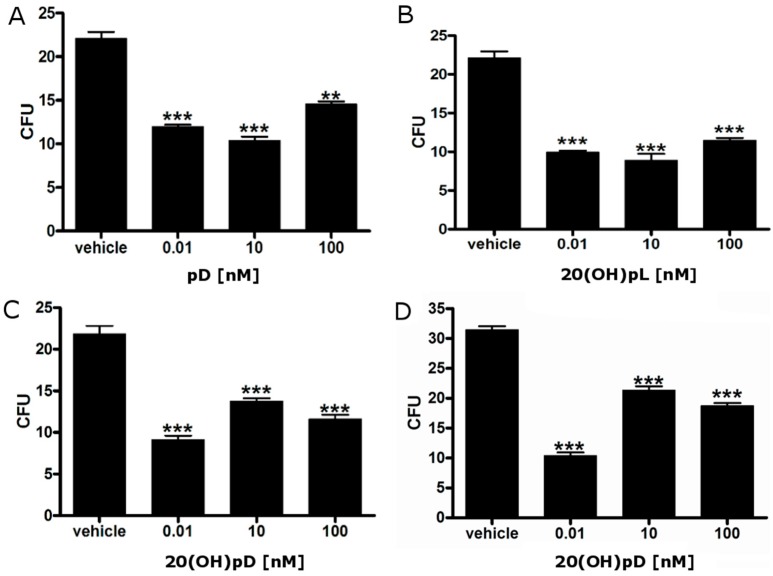
Short side-chain vitamin D analogs inhibit the ability of AbC1 hamster melanoma cells to form colonies in soft agar. Cells were grown in soft agar in the presence or absence of pD (**A**); 20(OH)pL (**B**); or 20(OH)pD (**C,D**). After three weeks, colonies with a diameter larger than 0.2 mm (**C**) or 0.5 mm (**D**) were counted. Data are shown as means ± SD (*n* = 4), statistical significance was estimated using t-test and presented as ** *p* < 0.01, *** *p* < 0.001.

We tested the inhibitory effects of Vitamin D and lumisterol analogs with a short side-chain, including pD, 20(OH)pL and 20(OH)pD, on colony formation in soft agar. All three analogs inhibited colony formation in soft agar at all concentrations tested (Figure 3). In addition, the detailed effect of 20(OH)pD on anchorage-independent growth, where colonies of different sizes (0.2 or 0.5 mm) were measured, is shown in Figure 3C,D.

### 2.3. 1,25(OH)_2_D_3_ Decreases the Number and Size of Colonies Formed by Hamster Ab Melanoma Cells Freshly Isolated from Solid Tumors

**Figure 4:**
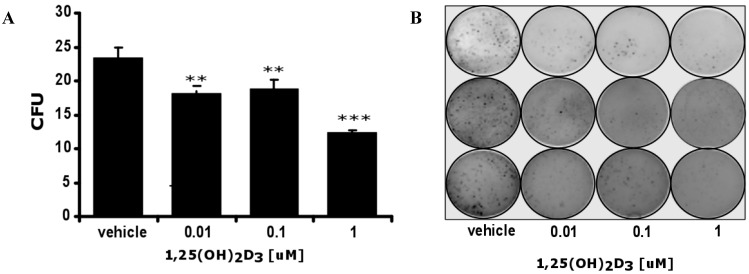
1,25(OH)_2_D_3_ inhibits Ab hamster melanoma colony formation in soft agar. Ab cells were grown in soft agar in the presence or absence of 1,25(OH)_2_D_3_. After three weeks, colonies were counted. Statistical significance was estimated using the t-test and data are presented as ** *p* < 0.01, *** *p* < 0.001, ±SD (**A**); Representative plate (*n* = 3) showing inhibition of Ab melanoma colony formation (**B**).

The AbC1 line represents a continuous line derived from Ab melanoma that has been propagated *in vitro* since 1987 [56]. Therefore, further detailed testing was performed on primary cell cultures using freshly isolated melanoma cells from transplantable Ab amelanotic tumors. 1,25(OH)_2_D_3_ decreased the ability of primary Ab cells to form colonies in soft agar assays, in a dose-dependent manner, and also decreased their size (Figure 4). Although the AbC1 cell line was isolated directly from an Ab hamster melanoma, it should be treated as a separate continuous cell line. Therefore, future *in vivo* studies on short side chain Vitamin D and lumisterol analogues are required to compare Ab melanoma sensitivity with classical 1,25(OH)_2_D_3_, although differences are suggested by studies on the AbC1 line (Figure 3 and Figure 4).

### 2.4. Expression of Vitamin D Related Genes Is Modulated by Melanin Pigmentation

**Figure 5:**
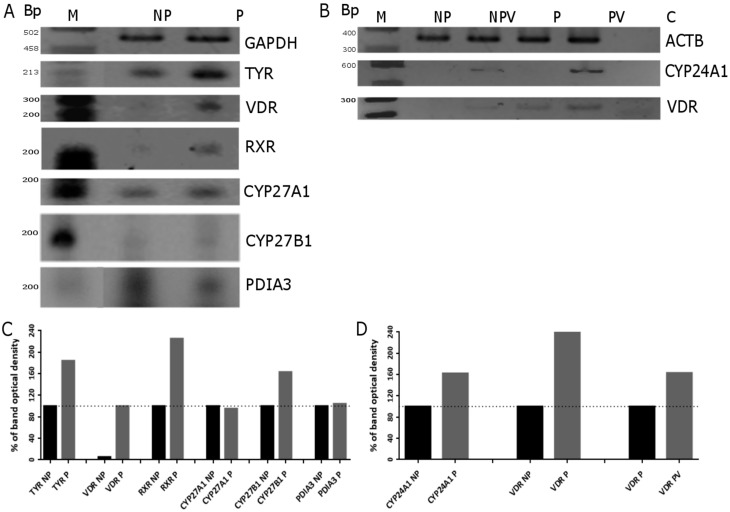
Melanogenesis and 1,25(OH)_2_D_3_ modulate the expression of tyrosinase (*TYR*), Vitamin D receptor (*VDR*), retinoid X receptor (*RXR*), disulfide isomerase (*PDIA3*), 25-hydoxylase (*CYP27A1*) and 1α-hydroxylase (*CYP27B1*) genes in hamster Ab melanoma cells. The effects of pigmentation (**A**) and 1,25(OH)_2_D_3_ on pigmented and non-pigmented cells (**B**) on the expression of selected genes were measured by semi-quantitative PCR. PCR analysis was performed as described in Materials and Methods. Glyceraldehyde 3-phosphate dehydrogenase (GAPDH) and β-actin (ACTB) were used as control genes. Length of amplicon fragments (in base pairs): GAPDH (496), Actin (353), TYR (205), VDR (234), RXR (210), CYP27A1(209), CYP27B1 (189), CYP24A1 (201). Densitometric analysis of PCR results (from panel A and B) obtained using TotalLab Quant v 11.4 (**C,D**). M, DNA marker; NP, non-pigmented cells (F10); NPV, non-pigmented cells treated with 1,25(OH)_2_D_3_; P, pigmented cells (DMEM); PV, pigmented cells treated with 1,25(OH)_2_D_3_; and C, control. Panels **C** and **D** showed quantification of band density from panels **A** and **B**, respectively.

As reported previously, media rich in tyrosine, such as DMEM, activates the pigmentary system in Ab hamster melanoma cells [57,58] with an accompanying increase in tyrosinase activity [59,60]. These results were confirmed in the current study (see inserts in Figure 1 and Figure 5). Since sensitivity of cells to Vitamin D depends on expression of several genes involved in regulation of its activity and metabolism, we investigated how production of melanin pigment influences their expression. It was shown that pigmentation dramatically increased the expression of VDR and its co-receptor RXR at the mRNA level (Figure 5A), while expression of 25-hydoxylase (*CYP27A1*) mRNA was only slightly elevated by pigmentation. Interestingly, expression of the potential alternative Vitamin D receptor *PDIA3* decreased in pigmented Ab cells. The increases in *VDR* and 24-hydroxylase *CYP24A1* mRNA levels after 1,25(OH)_2_D_3_ treatment were more pronounced in pigmented Ab cells than in non-pigmented ones (Figure 5B).

In order to confirm proper PCR amplification of fragments of hamster *TYR*, *PDIA3* and *CYP24A1* mRNAs, partial DNA sequencing of the PCR fragments was performed (Figure S1). *In silico* translation of *TYR*, *PDIA3* and *CYP24A1* PCR fragments, additionally confirmed the correct identification of the amplicons (Figure S2).

**Figure 6:**
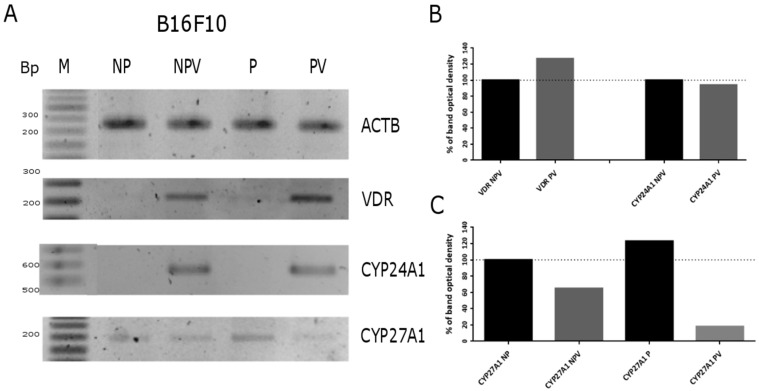
Melanogenesis and 1,25(OH)_2_D_3_ modulate *VDR, CYP24A1 and CYP27A1* gene expression in B16-F10 melanoma cells. 1,25(OH)_2_D_3_ was used at a concentration of 1 μM. PCR analysis was performed as described in Materials and Methods, β-actin (ACTB) was used as a control (**A**); The lengths of amplicons fragments (in base pairs) were: Actin (353), VDR (234), CYP24A1 (201), CYP27A1 (209). Densitometric analysis of PCR results obtained using TotalLab Quant v 11.4 (**B,C**). NP, non-pigmented cells (F10); NPV, non-pigmented cells treated with 1,25(OH)_2_D_3_; P, pigmented cells (DMEM); PV, pigmented cells treated with 1,25(OH)_2_D_3_. Data are from the most representative measurement.

**Figure 7:**
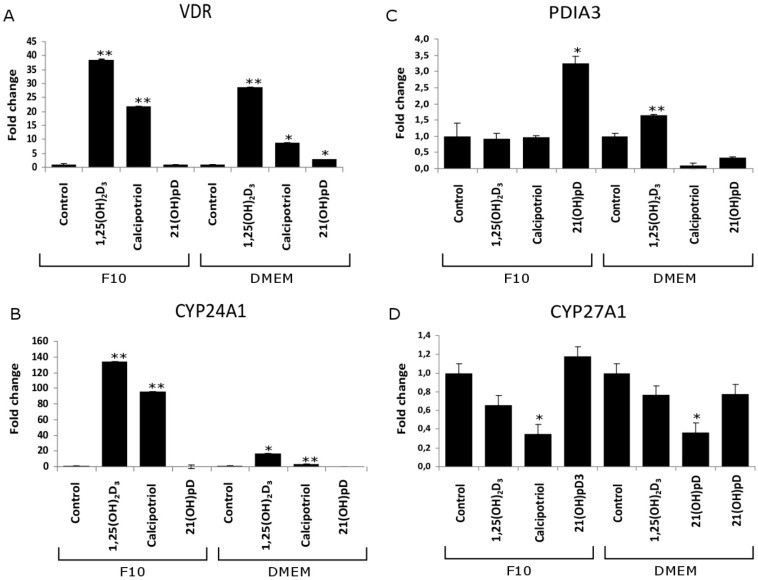
Influence of 1,25(OH)_2_D_3_, calcipotriol and 21(OH)pD on the expression of *VDR*, *PDIA3*, *CYP24A1* and *CYP27A1* genes in pigmented and non-pigmented B16-F10 melanoma cells. *VDR* (**A**); *PDIA3* (**B**); *CYP24A1* (**C**) and *CYP27A1* (**D**) mRNA levels were determined by quantitative PCR as described in Material and Methods. The expression of the genes was normalized by the comparative ΔΔ−*C*_t_ method, using β-actin as a housekeeping gene. Statistical significance was determined using the *t*-test and data are presented as * *p* < 0.05, ** *p* < 0.01 ±SD.

As shown in Figure 6, in murine B16-F10 cells, the switch of medium from F-10 (low tyrosine) to DMEM:F-10 mixture (50:50, high in tyrosine) initially stimulated expression of *VDR* mRNAs (moderate melanin pigmentation) but then it inhibited its expression when the cells became heavily pigmented (not shown). Administration of 1 μM 1,25(OH)_2_D_3_ to B16-F10 cells increased *VDR* and *CYP24A1* mRNA levels in both pigmented and non-pigmented melanoma cells (Figure 6). Unfortunately, quantification of band intensity of VDR and CYP24A1 PCR products was not possible due to lack of visible product in untreated cells (both pigmented and non-pigmented B16-F10). In the case of CYP27A1, its expression was slightly increased by pigmentation while treatment of pigmented B16-F10 cells with 1,25(OH)_2_D_3_ attenuated its expression. The relative levels of *VDR*, *CYP24A1*, *CYP27A1 and PDIA3* mRNA in control and ligand stimulated cells was analyzed further by real time PCR. Interestingly, the induction of the expression of *VDR* and its downstream *CYP24A1* genes by 1,25(OH)_2_D3 and calcipotriol was attenuated in pigmented cells in comparison to the non-pigmented ones (Figure 7A,B). For example, pigmented B16-F10 cells showed eight-fold lower *CYP24A1* expression after treatment with 1,25(OH)_2_D_3_ and a 25-fold lower expression after calcipotriol treatment, compared to non-pigmented cells (Figure 7B). In addition, 1,25(OH)_2_D_3_ stimulated expression of *VDR* and *CYP24A1* genes to a higher degree than calcipotriol in both pigmented and non-pigmented cells (Figure 7A,B). Interestingly, treatment of both pigmented and non-pigmented B16-F10 cells with 1,25(OH)_2_D_3_ and calcipotriol resulted in reduction of *CYP27A1* expression and the effect was more pronounced in cells treated with calcipotriol than with 1,25(OH)_2_D_3_ (Figure 7C).

Treatment with 21(OH)pD, a short side-chain analog of Vitamin D (36), increased *PDIA3* mRNA levels in non-pigmented B16-F10 cells (Figure 7D), but had no effect on *VDR*, *CYP27A1* or *CYP24A1* mRNA. However, mRNA levels of *PDIA3* decreased in pigmented B16-F10 cells treated with calcipotriol and 21OHpD, while slightly increasing in cells treated with 1,25(OH)_2_D_3_ (Figure 7C).

### 2.5. VDR Receptor Translocation to the Nucleus Is Stimulated by 1,25(OH)_2_D_3_

**Figure 8:**
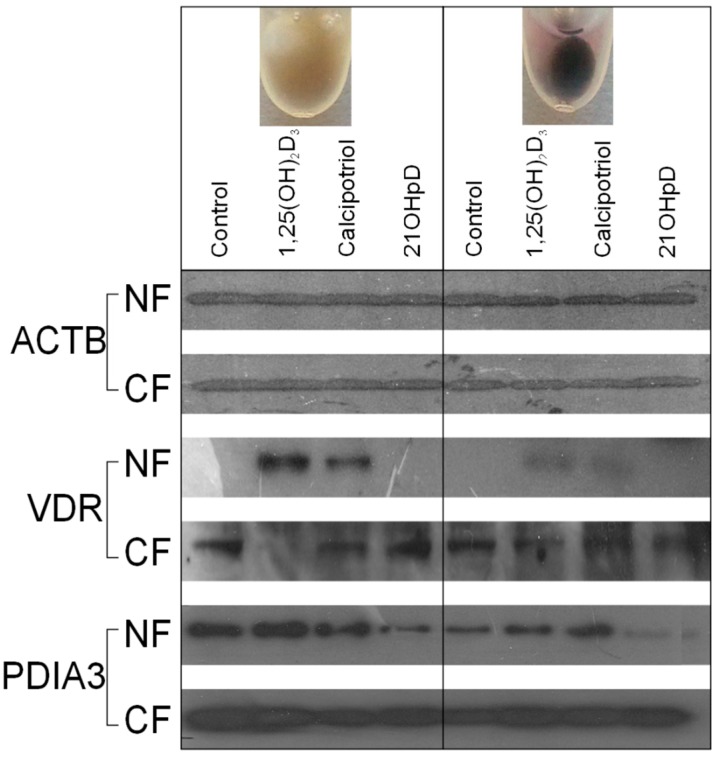
Western blot analysis of the effects of 1,25(OH)_2_D_3_, calcipotriol and 21(OH)pD on the cytoplasmic and nuclear location of the VDR and the PDIA3 receptor. Pigmented and non-pigmented B16-F10 melanoma cells were treated with 10 nM 1,25(OH)_2_D_3_, calcipotriol or 21(OH)pD, and VDR and PDIA3 receptors in cytoplasmic (CF) and nuclear fractions (NF) measured by western blotting, as described in the Materials and Methods section. Actin (ACTB) was used as a loading control.

Western blotting analysis showed that the VDR is synthesized in B16-F10 mouse melanoma cells and its translocation to the nucleus is stimulated by treatment with 1,25(OH)_2_D_3_ or calcipotriol (Figure 8). Interestingly, ligand induced translocation of the VDR to the nucleus is attenuated in pigmented cells in comparison to the non-pigmented ones. However, 21(OH)pD had no effect on VDR protein expression nor its translocation to the nucleus. In addition, Western blot analysis demonstrated a negative correlation between 21(OH)pD treatment and the amount of PDIA3 in the nuclear fraction, while nuclear localization of PDIA3 was slightly increased by 1,25(OH)_2_D_3_ or by calcipotriol treatment of pigmented B16-F10 melanoma cells.

## 3. Discussion

In this paper, we report the differential anti-proliferative responses of pigmented and non-pigmented rodent melanoma cells to classical metabolites of Vitamin D (1,25(OH)_2_D_3_ and 25(OH)D_3_) and novel 20(OH)D_3_ [61] and calcipotriol [62], as well as short-side chain secosteroids, pD [63], 20(OH)pD, 20(OH)pL [64] and 21(OH)pD [36]. It should be noted that 20(OH)D_3_, pD, 20(OH)pD and 21(OH)pD are non- or low- calcemic secosteroids that may represent therapeutic alternatives to 1,25(OH)_2_D_3_, which is highly calcemic [32,33,34,35,36,37,38,39,40,41,42,43,44]. These compounds belong to a new class of endogenously produced Vitamin D analogs, with their 5,7-diene-like precursors potentially being made in the skin or other tissues [34,40,41,42,65]. Furthermore, formation of 21(OH)pD from 21(OH)7DHP occurs in skin subjected to UVB irradiation under *ex vivo* conditions [36]. Interestingly, the short side-chain lumisterol-like derivative, 20(OH)pL, which is generated together with 20(OH)pD after UVB irradiation of 20(OH)7DHP [36], was also found to inhibit murine melanoma cell growth, broadening the spectrum of biologically active photo-derivatives of 5,7-dienes.

An additional goal of this study was to characterize the effects of inducible pigmentation on the response to Vitamin D analogs in two melanoma cell lines, murine B16-F10 and hamster Ab. Both cell lines are fully synergistic with animal models (mouse and hamster, respectively), enabling their future direct use in animal studies.

Production of melanin has been shown to influence Vitamin D production in the skin [14]. In human melanoma cells, melanin pigment attenuates their responsiveness to gamma radiation, the cytotoxic effects of chemotherapeutics and immunotoxic activities towards tumor cells [52,53,54]. Melanogenesis also decreases the sensitivity of human melanoma cells to treatment with Vitamin D derivatives *in vitro* [46]. Finally, pigmentation of human melanomas *in vivo* shows a reverse correlation with the expression of VDR [50,66] and CYP27B1 [67]. These studies indicate that melanin pigmentation can impair both the metabolism and activity of classical Vitamin D derivatives in human melanomas.

The sensitivity of murine B16-F10 melanoma to Vitamin D analogs has been widely investigated with contradictory results, with the responsiveness to the treatment usually being related to VDR expression (see for review and discussion: [35]). Current studies on rodent melanomas show that expression of VDR is below the level of detectability in non-pigmented cells by conventional PCR. However, treatment with 1,25(OH)_2_D_3_ and calcipotriol resulted in a significant increase in the level of the VDR transcript, with 1,25(OH)_2_D_3_ being the more effective of the two secosteroids (Figure 5, Figure 6, and Figure 7). The increased expression of VDR transcript was observed in both pigmented and non-pigmented cells and correlated with induction of the expression of its main downstream target gene, *CYP24A1*. Interestingly, induction of melanin pigmentation attenuated 1,25(OH)_2_D_3_ and calcipotriol induced expression of *VDR* and *CYP24A1* genes (Figure 7A,B). This finding is consistent with attenuation of 1,25(OH)_2_D_3_ induced translocation of VDR to the nucleus in pigmented melanoma cells (Figure 8), which again explains the reduction by eight-fold of the stimulation of *CYP24A1* gene expression by 1,25(OH)_2_D_3_ (Figure 7B). In contrast, treatment with the short side-chain Vitamin D analog, 21(OH)pD, did not stimulate expression of either VDR or CYP24A1 mRNAs. Based on the presented data, we suggest that very low expression of VDR is sufficient for activation of Vitamin D genomic responses, and VDR itself is one of the first targets. Various mechanisms may trigger VDR expression with the induction of VDR-regulated genes being a secondary effect.

Our data indicate that moderate pigmentation sensitizes murine melanoma cells to Vitamin D analogs, including 1,25(OH)_2_D_3_, 25(OH)D_3_, calcipotriol and 21(OH)pD. The inhibitory effect of 1,25(OH)_2_D_3_ on proliferation is consistent with previous reports (for review see [19,34,35]). For example, an increased sensitivity of pigmented human SKMEL-188 melanoma cells towards 1,25(OH)_2_D_3_, 21(OH)pL, 21(OH)7DHP or its oxidized analog 21(OH)oxy-piT was shown recently [36]. In the same study, the activity of 21(OH)pD was not affected by pigmentation. In the current study, pigmented murine B16-F10 cells were only slightly more sensitive than non-pigmented ones. This response contrasted with that of pigmented hamster Ab melanoma cells, which were several fold more sensitive to 21(OH)pD than non-pigmented cells. Interestingly, an induction of heavy pigmentation in human SKMEL-188 cells attenuated the anti-proliferative effects of 20(OH)D_3_, which was accompanied by decreased expression of VDR at the protein level [46]. With B16-F10 murine melanoma in the current study, 20(OH)D_3_ exerted an inhibitory effect only at the highest concentration tested (10^−6^ M) in both pigmented and non-pigmented cells. These data indicate that the sensitivity of melanoma cells to Vitamin D derivatives can be influenced by the intensity of melanin pigmentation in a species-, cell type- and condition-dependent manner. Furthermore, it can be speculated that production of pigment influences the metabolism and stability of Vitamin D analogs, and thus this phenomenon requires further in depth investigation. However, observed changes in the level of CYP27A1 mRNA may suggest enhanced 25-hydoxylase activity. Interestingly, production of melanin is also accompanied by reactive oxygen species (ROS) generation. Although the presence of ROS is usually linked to Vitamin D degradation, recently we described a new oxidized photo-derivative of 21(OH)7-dehydropregnonolene (21(OH)oxy-piT) as a potent inhibitor of human SKMEL-188 melanoma proliferation [36].

The treatment of murine B16-F10 cells with 21(OH)pD caused antiproliferative effects, but did not lead to an increase in *VDR* or *CYP24A1* mRNA levels. It is well established that increased CYP24A1 expression leads to increased inactivation of Vitamin D analogs [68], thus the high *CYP24A1* mRNA expression after treatment may, at least partially, explain the observed limited growth inhibition of non-pigmented mouse B16-F10 cells. It has to be noted that short side-chain analogs, such as 21(OH)pD, are not a target for 24-hydroxylation because they lack a sterol side-chain with C24. The lack of stimulation of CYP24A1 expression in murine B16-F10 melanoma cells by 21(OH)pD may be explained by the impaired interaction with the VDR. Indeed, our study shows that the secosteroids tested do differ in their ability to induce the translocation of the VDR into the nucleus of B16-F10 cells. In comparison to 1,25(OH)_2_D_3_, calcipotriol showed only partial activity, while treatment with 21(OH)pD did not result in any detectable nuclear localization of the VDR. Recent molecular modeling studies by Kim *et al*. [69], indicated that a range of Vitamin D analogs differed in their predicted VDR binding capacity in a manner that correlated with their ability to stimulate VDR translocation to the nucleus. According to this *in silico* modeling, 21(OH)pD showed the lowest VDR docking score (weakest binding) among the Vitamin D analogs tested [69], and this corresponded to a poor ability to translocate the VDR from the cytoplasm to the nucleus [36,69]. In contrast to murine B16-F10 cells, treatment of human SKMEL-188 melanoma cells with 21(OH)pD resulted in partial translocation of VDR to the nucleus [36,69], suggesting possible species- or cell line-dependent differences in the response. Thus, the effects of pigmentation on the activity of Vitamin D analogs appear to depend not only on VDR expression and its translocation to the nucleus, but also on the structural properties of the secosteroid and possibly other cell-specific factors or receptors yet to be identified. The antiproliferative potential of 21(OH)pD, in conjunction with its low-calcemic activity and limited requirement for genomic activity of the VDR, warrants further *in vivo* testing against advanced, pigmented melanomas. This secosteroid could be of great value for the treatment of melanomas which display decreased expression of the VDR and enhanced expression of CYP24A1.

It has been suggested that PDIA3 (ERp57) is a Vitamin D-binding protein, which could also act as a transcription factor after stimulation [70,71]. The effects of Vitamin D analogs on PDIA3 mRNA levels and translocation to the nucleus were tested in B16-F10 cells. Only treatment with 21(OH)pD resulted in an elevated level of *PDIA3* mRNA in non-pigmented cells, while 21(OH)pD and calcipotriol decreased *PDIA3* mRNA expression in pigmented cells. 1,25(OH)_2_D_3_ had only a minor, but statistically significant, stimulatory effect on the *PDIA3* mRNA level in pigmented B16-F10 cells. Interestingly, while 1,25(OH)_2_D_3_ and calcipotriol had only minor effects on the translocation of PDIA3 to the nucleus, 21(OH)pD inhibited nuclear translocation in both pigmented and non-pigmented B16-F10 cells. Therefore, PDIA3 might be considered as an alternative target for analogs with a short side-chain but the mechanism of their action requires further studies. In addition, it remains to be tested whether retinoic acid orphan receptors (RORs) can serve as a target for 21(OH)pD, since Vitamin D analogs with a full-length side chain affect the activity of RORα and γ [72].

When analyzing the melanin pigmentary system, it is important to note that an enhanced inhibitory effect of Vitamin D analogs is only observed immediately after induction of melanogenesis, *i.e.*, in mildly pigmented melanoma cells. Prolonged exposure to high levels of tyrosine resulted in heavy pigmentation of both Ab and B16-F10 melanoma cells, followed by inhibition of proliferation and cell detachment. Hyperpigmentation of melanoma cells is also associated with a decrease of the *VDR*, *RXR* and *PDIA3* mRNA levels in B16-F10 cells. Furthermore, pigmented B16 melanoma cells show attenuation of responsiveness to ligand stimulation of VDR translocation to nucleus (Figure 8), and expression of *VDR* and *CYP24A1* genes (Figure 7A,B), indicating that VDR signaling is malfunctioning in melanotic cells. These findings are consistent with a report showing that a high level of pigmentation of human melanomas was correlated with low expression of the VDR and CYP27B1 *in vivo* [50,67] and downregulation of VDR protein expression in pigmented human melanma line [50,66]. Since pigmentation affected overall disease-free survival in melanoma patients [49], further studies on the complex relationship between Vitamin D response, pigmentation and melanoma behavior are mandatory.

## 4. Materials and Methods

### 4.1. Cell Lines and Vitamin D Analogs

The Bomirski hamster Ab melanoma line originated in 1963 from the transplantable Ma melanotic melanoma of the Syrian hamster, through a process of tumor progression that included an increase in the growth rate, loss of melanin pigmentation and significant shortening of the overall survival of animals bearing the melanomas [73]. Since then, the Ab hamster melanoma line has been maintained *in vivo* by consecutive subcutaneous transplantations of tumor material [73,74,75]. The hamster Ab melanoma cells were obtained for each experiment from solid tumors using a non-enzymatic method of cell isolation, as previously described [76]. The resection of the tumor was performed after 10–11 days of growth after the transplantation. The cell suspensions used in the experiments consisted of 95%–98% viable cells, as estimated by the trypan blue exclusion assay. The transplantation procedure was approved by the Animal Ethics Committee at the Medical University of Gdansk and was in accordance with the National Health and Medical Research Council’s guide for the care and use of laboratory animals. Experiments were performed on freshly isolated cells defined as a primary melanoma cell culture.

Hamster Ab primary cells and the mouse B16-F10 cell line were cultured, in RPMI and Ham’s F-10 media, respectively, supplemented with 10% fetal bovine serum (Sigma, Poznan, Poland) and antibiotic-antimycotic solution (Sigma, Poznan, Poland). Culturing Ab melanoma cells in a medium high in tyrosine induced rapid melanin production with attendant changes in the differentiation program of the cells [57,58]. The tyrosine concentrations in media used were: DMEM, 423 μM; F-10, 11 μM; 50:50 DMEM:F-10, 217 μM; RPMI, 118 μM; 50:50 DMEM:RPMI, 270 μM.

The hamster AbC1 melanoma cell line, cloned from a primary culture of Bomirski Ab melanoma [56], was cultured in Ham’s F-10 medium supplemented with glucose, L-glutamine, pyridoxine hydrochloride (Cellgrow, Manassas, VA, USA), 5% fetal bovine serum (FBS) (Sigma, Poznan, Poland) and 1% penicillin/streptomycin/amphotericin antibiotic solution (Sigma, Poznan, Poland), as described previously [56,77]. For experimental purposes, 5% charcoal-stripped FBS (GE Healthcare Life Sciences, Warsaw, Poland) was used. The AbC1 cell line is an excellent model for pigmentation studies, as increasing the concentration of tyrosine in the medium induces melanin pigmentation of these cells and changes their biological activities [56,75,78,79].

**Figure 9:**
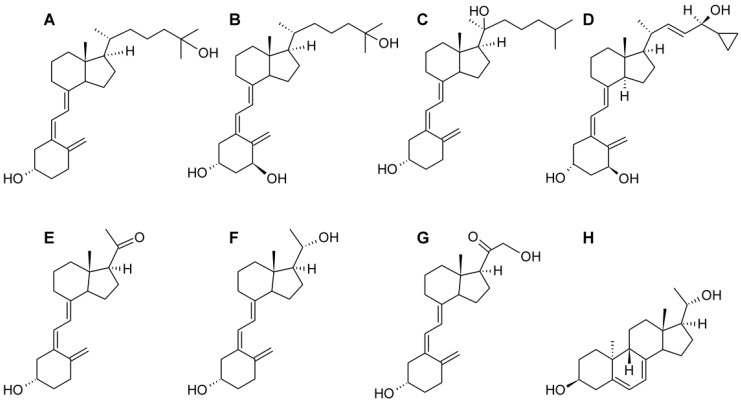
Chemical structures of the Vitamin D analogs tested. (**A**) 25(OH)D_3_; (**B**) 1,25(OH)_2_D_3_; (**C**) 20(OH)D_3_; (**D**) calcipotriol; (**E**) pD; (**F**) 20(OH)pD; (**G**) 21(OH)pD; (**H**) 20(OH)pL.

1,25(OH)_2_D_3_, 25(OH)D_3_ and calcipotriol were acquired from Pharmaceutical Research Institute, Warsaw, Poland. Other Vitamin D analogs: pD, 20(OH)pD, 20(OH)pL and 21(OH)pD were synthesized and purified as described previously [36,64]. 20(OH)D_2_ and 20(OH)D_3_ analogs were also synthesized and purified as described previously [80,81,82,83]. The chemical structures of these secosteroids are presented in Figure 9.

### 4.2. MTT Assay

Hamster melanoma cells were seeded at a density of 8000 cells per well into 96-well plates in RPMI or a mixture of RPMI:DMEM (50:50, *v*/*v*) media (Sigma, Poznan, Poland), supplemented with 2% charcoal-stripped fetal bovine serum (Biological Industries, Israel) and antibiotic-antimycotic solution (Sigma, Poland). After incubation for 24 h, the medium was replaced with a fresh one containing serial dilutions of 1,25(OH)_2_D_3_ or its analogs (at 0.01–1000 nM concentrations). After incubation for 48 h, 20 μL of MTT (3-(4,5-dimethylthiazol-2-yl)-2,5-diphenyltetrazoliumbromide; 5 mg/mL in PBS; Sigma, Poland) were added to each well and the plates were incubated for a further 3 h. Media were discarded and 100 μL of acidified isopropanol (containing 0.1 M hydrochloric acid) were added. The absorbance was measured at 570 nm.

### 4.3. SRB Assay

B16-F10 cells were seeded at a density of 8000–10,000 per well in 96-well plates in F-10 or DMEM medium (Sigma, Poland), supplemented with 2% charcoal-stripped fetal bovine serum (Biological Industries, Israel) and antibiotic-antimycotic solution (Sigma, Poznan, Poland). After incubation for 24 h, medium was replaced with a fresh one containing vehicle, 1,25(OH)_2_D_3_ or its analogs at concentrations of 1 pM to 1 μM. Following incubation for 48 h, 100 μL of 20% TCA were added and cells incubated for 1 h in 4 °C. The medium was then removed and 100 μL of SRB (sulforhodamine B) were added and the plates incubated for 15 min at room temperature. The SRB was discarded, cells washed and 150 μL of 10 mM Tris (pH 10.5) were added. The absorbance was measured at 570 nm.

### 4.4. PCR-Based Expression Analysis

RNA was isolated using a Total RNA kit (A&A Biotechnology, Gdynia, Poland). Reverse transcription (500 ng RNA/reaction) was performed with a First Strand cDNA Synthesis Kit (Fermentas, Lithuania). PCR reactions were performed using cDNA (diluted 10-fold in sterile water) and PCR MIX PLUS (A&A Biotechnology, Gdynia, Poland). Quantitative PCR analysis was performed using cDNA (diluted 5-fold in sterile water) and 2× PCR Master Mix SYBR (A&A Biotechnology). The primers for both lines were designed based on the mice and rat sequences using Primer Quest software (Integrated Device Technology, San Jose, CA, USA). The primers for the housekeeping gene, GAPDH, were obtained from the First Strand cDNA Synthesis Kit (Fermentas, Lithuania). The sequences of the primers are shown in Table S1. Standard PCR reactions were performed using a MJ Mini Bio-Rad cycler. Following agarose gel electrophoresis, the amplicons were visualized by ethidium bromide staining. For Ab cells, PCR fragments encoding tyrosinase, PDIA3 and CYP24A1 were sequenced by the Institute of Biochemistry and Biophysics (Polish Academy of Science, Warsaw, Poland). The resulting DNA sequences were compared with mouse and rat genes by multiple alignment algorithm using ClustalW2 software (Figure S1). After *in silico* translation of Ab sequenced fragments, predicted sequences of hamster proteins were aligned with the corresponding mouse and rat protein sequences (Figure S2).

### 4.5. Colony Formation Assay

The ability of hamster melanoma cells to form colonies in soft agar, as an equivalent of tumorigenicity, was determined as described previously [45], with some modifications. The bottom of the wells of a 12-well plate was covered with 0.5 mL of solubilized 0.8% agar solution in RPMI medium (base-mix). After solidification of the base, a top-mix of 0.4% agar solution in RPMI medium containing 500 Ab Bomirski cells or 1000 AbC1 cells, was placed on the top. The solidified soft agar cell layer was covered with 100 μL of liquid RPMI medium containing serial dilutions of Vitamin D and its analogs. 1,25(OH)_2_D_3_ was used at final concentrations of 10 nM, 100 nM or 1 µM for Ab cells, while 20(OH)pD, pD and 20(OH)pL were tested at concentrations of 0.01 nM, 10 nM or 100 nM, and 100 µM for AbC1 cells. Cells were grown under 5% CO_2_ at 37 °C for 2–3 weeks with the 100 µL of medium containing the secosteroids being changed every 2–3 days. Ab melanoma colonies were identified using ethidium bromide (0.2 mL/well) and visualized under a UV transilluminator. AbC1 melanoma colonies were stained with 500 µL/well of MTT (0.5 mg/mL) (Promega, Mannheim, Germany). Colonies were photographed and counted using ImageJ software.

### 4.6. Western Blotting

Pigmented and non-pigmented B16-F10 cells were treated for 24 h with 10 µM 1,25(OH)_2_D_3_, calcipotriol ((1*S*,3*R*,5*Z*,7*E*,22*E*,24*S*)-26,27-Cyclo-9,10-secocholesta-5,7,10,22-tetraene-1,3,24-triol) or 21(OH)pD. The Nuclear Extract Kit (Active Motif, La Hulpe, Belgium) was used for preparation of nuclear and cytoplasmic fractions. The protein concentration was measured by the Bradford method and samples were stored at −80 °C. The detection of VDR, PDIA3 and actin in nuclear and cytoplasmic extracts was performed using previously described methodology (46). Anti-VDR (1:100, Abcam, Cambridge, Great Britain), anti-PDIA3 (1:250, Sigma-Aldrich, Poznan, Poland) and anti-AKTB (1:200, Santa Cruz Biotechnology, CA, USA) antibodies were used to detect the antigens. The appropriate secondary antibody coupled to horseradish peroxidase (1:2000, Santa Cruz Biotechnology, Heidelberg, Germany) was then applied and proteins were visualized using Super Signal West Pico (Pierce Biotechnology, Rockford, IL, USA).

## 5. Conclusions

Our study suggests that complex mechanisms are involved in the antiproliferative activity of Vitamin D analogs on melanoma cells, which include a modulatory role of melanin pigmentation. First, low calcemic analogs of Vitamin D are potent inhibitors of rodent melanoma cells proliferation; Second, mildly pigmented melanoma cells are more sensitive to several Vitamin D analogs than non-pigmented ones; Third, pigmented cells not only show attenuation of ligand-induced translocation of VDR to the nucleus but also ligand-induced expression of *VDR* and *CYP24A1* genes is reduced in comparison to non-pigmented cells; Last, nuclear localization of VDR and activation of the classical (genomic) Vitamin D response pathway may not be necessary for growth inhibition of murine cells. Other mechanisms should be taken into consideration, including but not limited to involvement of Vitamin D analogs in the regulation of PDIA3, Wnt/β-catenin, Hedgehog, Notch, RORα and RORγ related pathways [35,71,84]. We also speculate that elevated levels of reactive oxygen species (ROS) associated with active melanogenesis may stimulate formation of oxidized derivatives of Vitamin D analogs thereby modulating their activity. A similar effect was shown recently following prolonged UVB irradiation of 21-hydroxydehydropregnenolone, the 21(OH)pD precursor [36]. Thus, active melanogenesis may decrease the potency of some Vitamin D analogs by ROS-mediated deactivation while melanin, by sequestrating Vitamin D compounds, may reduce their availability to interact with the corresponding receptors. This hypothesis requires further testing.

## Supplementary Materials

Supplementary materials can be found at http://www.mdpi.com/1422-0067/16/04/6645/s1.
