# Supplementary material for: Antitumor Effects of Vitamin D Analogs on Hamster and Mouse Melanoma Cell Lines in Relation to Melanin Pigmentation
Source: Int J Mol Sci. 2015 Mar 24;16(4):6645–67. doi: 10.3390/ijms16046645 (PMC4424981; doi:10.3390/ijms16046645)
Supplement: Supplementary file 1 [file ijms-16-06645-s001.pdf]

Supplementary Information

TYR

```
Ab      ATGACAAA--CCAAAACCCCGAGGCTCCCATCTTCGGCAGATGTGGAATTTTGTCTGACTT 59
Mus     ATGACAAAGCCAAAACCCCCAGGCTCCCATCTTCAGCAGATGTGGAATTTTGTCTGAGTT 60
Rattus  ATGACAAAGCCAAAACCCCCAGGCTCCCATCTTCAGCAGACGTGGAATTTTGTTTGAGTT 60

Ab      TAACCCAGTATGAAGCTGGCTCCATGGATAAAACTGCCAATTTCAGCTTTAGAAACACAC 119
Mus     TGACCCAGTATGAATCTGGATCAATGGATAGAACTGCCAATTTCAGCTTTAGAAACACAC 120
Rattus  TGACCCAGTATGAATCTGGATCAATGGATAGAACTGCCAATTTCAGCTTTAGAAACACAC 120

Ab      TGGAAGGATTTGCAAATCCACTCACAGGGATAGCAGAA 157
Mus     TGGAAGGATTTGCCAGTCCACTCACAGGGATAGCAGAT 158
Rattus  TGGAAGGATTTGCCAGTCCACTCACAGGGATAGCAGAT 158
```

MusmusculusGenBank: AK014619.1
RattusNCBI Reference Sequence: NM_001107535.1

PDIA3

```
Ab      GGGCTCATGCTAGTCGAGTTCTTCGCCCCTTGGTGTGGACATTGCAAGAGGCTTGCCCCT 60
Mus     GGGCTCATGCTAGTCGAGTTCTTCGCCCCCTGGTGTGGACATTGCAAGAGGCTTGCCCCT 60
Rattus  GGGCTCATGCTAGTCGAGTTCTTCGCCCCATGGTGTGGACATTGCAAGAGGCTTGCCCCT 60

Ab      GAGTATGAAGCTGCAGCAA 79
Mus     GAGTATGAAGCTGCAGCAA 79
Rattus  GAGTATGAAGCTGCAGCAA 79
```

MusMusculus GenBank: BC003285.1
Rattus GenBank: DY311951.1

CYP24A1

```
Ab      AAAGAACTATATGCTGCGGTCACTGAGCTCCAGCTTGCTGCAGTGGAGACGACAGCCAAC 60
Mus     AAAGAACTGTACGCTGCTGTCACGGAGCTCCAGCTGGCTGCAGTGGAGACGACCGCAAAC 60
Rattus  AAGGAACTGTACGCCGCTGTCACGGAGCTGCAGCTCGCTGCAGTGGAGACGACCGCGAAC 60

Ab      AGCCTGATGTGGGTTCTCTACAACCTATGCCGGAATCCCCAAGTGCAGCAGAGACTTCTC 120
Mus     AGCTTGATGTGGATTCTCTACAATCTATCCCGGAATCCCCAAGTGCAACAGAGACTTCTC 120
Rattus  AGCTTGATGTGGATTCTCTACAATCTATCCCGGAATCCTCAAGCGCAACGGAGACTCCTT 120

Ab      CTGGAAATCCAGAGCGTGCTACCCGGGAACCAGATGCCACGGGCAGAAGACGTGAAGAAT 180
Mus     CGGGAAATCCAGAGCGTGCTGCCTGACAACCAGACGCCACGGGCGGAAGATGTGAGGAAT 180
Rattus  CAGGAAGTTCAGAGCGTGCTGCCTGACAATCAGACGCCACGGGCGGAAGACCTGAGGAAT 180

Ab      ATGCCCTATTTAAAGGCCTG 200
Mus     ATGCCCTATTTAAAGGCCTG 200
Rattus  ATGCCCTATTTAAAGGCCTG 200
```

MusMusculus GenBank: AK143984.1
Rattus NCBI Reference Sequence: NM_201635.2

Figure S1. Comparison of fragments of coding sequences of the hamster (*Mesocricetus auratus*) tyrosinase (TYR), disulfide isomerase (PDIA3) and 24-hydroxylase (CYP24A1) genes, obtained after isolation of the PCR fragments isolated from the gel and sequencing. Newly acquired hamster sequences were compared with the DNA sequences of mice (*Mus musculus*) and rat (*Rattus norvegicus*) from the NCBI database and Gen Bank *Mus musculus* GenBank AK014619.1, *Rattus norvegicus* NCBI NM_001107535.1; *Mus musculus* GenBank BC003285.1, *Rattus norvegicus* GenBank DY311951.1; *Mus musculus* GenBank: AK143984.1, *Rattus norvegicus* NCBI NM_201635.2. A 157-bp PCR fragment of the hamster *TYR* gene shared 92% identity with the mouse gene and 91% with the rat gene. A 79-bp PCR fragment of the hamster *PDIA3* gene shared 98% identity with both mouse and rat genes. A 200-bp PCR fragment of the hamster *CYP24A1* gene shared 89% identity with the mouse gene and 84% with the rat gene.

TYR

Ab MTNQNPEAPIFGRCGILSDFGRICKSTHRDSRITQYEAGSMDKTANFSFRNT 52
Mus QSQNPQAPIFSRCGILSEFGRICQSTHRDSRLTQYESGSMDRTANFSFRNT 51

MusMusculus GenBank: AAA40516.1

PDIA3

Ab GLMLVEFFAPWCGHCKRLAPEYEAAA 26
Mus GLMLVEFFAPWCGHCKRLAPEYEAAA 26

Mus Musculus NCBI Reference Sequence: NP_031978.2

CYP24A1

Ab KELYAAVTELQLAAVETTANSLMWILYNLSRNPQVQQRLLREIQSVLPDNQTPRAEDVRN 60
Mus KELYAAVTELQLAAVETTANSLMWILYNLSRNPQVQQRLLREIQSVLPDNQTPRAEDVRN 60

Ab MPYLKA 66
Mus MPYLKA 66

Mus musculus NCBI Reference Sequence: NP_034126.1

Figure S2. Comparison of fragments of the predicted proteins of Syrian hamster (*Mesocricetus auratus*) and mouse (*Mus musculus*). Sequenced DNA fragments of the tyrosinase (Tyr), disulfide isomerase (Pdia) and 24-hydroxylase (Cyp24a1) genes were translated *in silico* and compared with the corresponding mice proteins deposited in the NCBI gene Bank with associated references as shown: Mus musculus GenBank AAA40516.1; Mus musculus NCBI Reference Sequence: NP_031978.2; Mus musculus NCBI Reference Sequence: NP_034126.1. *In silico* translation of *TYR*, *PDIA3* and *CYP24A1* showed that their partial protein sequences shared, 82%, 100% and 100% identity, respectively, with the corresponding murine sequences.

Table S1. Sequences of PCR primers used in the study.

Primers	F:	R:
B-Actin	GCTCGTCGTCGACAACGGCTC	CAAACATGATCTGGGTCATCTTCT
TYR	TATGCGATGGAACACCTGAG	TCTGCTATCCCTGTGAGTGG
VDR	GCTGATCGAACCCCTCATAA	TTCTGGATCATCTTGGCGTA
RXR	AGGATATCAAGCCGCCACTA	TGTTGTCTCGGCAGGTGTAG
CYP27A1	TCTCTACCACCTTGCCTTGG	TGTTATCCCAGCCATTCAGG
CYP27B1	GCTCCTGCGACAAGAAAGTC	TCCAGAGTTCCAGCATAGCC
CYP24A1	TCAGCAGCCTAGTGCAGATTTCCC	GTCTTCACTGGATCCCAACACCTG
Pdia3	CTCCGATGTGTTGGAACTGA	CAGGTGTTTGTGTTGGCAGT
